# Accuracy of Dental Implant Placement by a Novel In-House Model-Free and Zero-Setup Fully Guided Surgical Template Made of a Light-Cured Composite Resin (VARO Guide^®^): A Comparative In Vitro Study

**DOI:** 10.3390/ma14144023

**Published:** 2021-07-19

**Authors:** Young Woo Song, Jongseung Kim, Jang-Hyun Kim, Ji-Man Park, Ui-Won Jung, Jae-Kook Cha

**Affiliations:** 1 Department of Periodontology, Research Institute for Periodontal Regeneration, Yonsei University College of Dentistry, Seoul 03722, Korea; tigger09@yuhs.ac (Y.W.S.); jseungkim@yuhs.ac (J.K.); drjew@yuhs.ac (U.-W.J.); 2 Department of Oral Medicine, Infection, and Immunity, Harvard School of Dental Medicine, Boston, MA 02115, USA; 3 Department of Prosthodontics, Yonsei University College of Dentistry, Seoul 03722, Korea; 881004kjh@yuhs.ac (J.-H.K.); jimarn@yuhs.ac (J.-M.P.)

**Keywords:** digital dentistry, virtual surgical planning, guided implant surgery, intraoral scan, cone beam computed tomography

## Abstract

Background: This in vitro study mainly aimed to compare VARO Guide^®^ to the surgical guide fabricated by CAD/CAM (NAVI Guide^®^) in terms of accuracy and efficacy of the implant surgery held in the dentiform model. Methods: Twenty surgeons, 10 dentists in the beginner group and 10 dentists in the expert group, participated in the study. Each surgeon conducted fully guided surgery in dentiform models twice, once with VARO Guide^®^ (VG surgery) and the other time with a conventional type of templates, NAVI Guide^®^ (NG surgery). Based on the superimposition of presurgical and postsurgical STL files, the positional deviations between the virtually planned and actually placed implants and the time spent on presurgical preparation and surgical procedures were estimated and compared. Results: All dimensional deviations were similar between the two groups (*p* > 0.05), and there was no significant difference between the expert and beginner groups regardless of the guide system. The total procedure time (mean (median)) of the VG surgery (26.33 (28.58) min) was significantly shorter than that of the NG surgery (378.83 (379.35) min; *p* < 0.05). While the time spent only for the fully guided implant surgery (from the start of the surgical guide sitting onto the dentiform model to the final installation of the implant fixture) was comparable (*p* > 0.05), the presurgical preparation time spent on virtual implant planning and surgical guide fabrication in the VG surgery (19.63 (20.93) min) was significantly shorter compared to the NG surgery (372.93 (372.95) min; *p* < 0.05). Conclusions: Regardless of experience, both VG and NG surgery showed reliable positional accuracy; however, the total procedure time and the preparation time were much shorter in the VG surgery compared to the NG surgery.

## 1. Introduction

With the advances in implant surface treatment, clinicians hardly suffer from failures after implant surgery [1], and now they seek to improve accuracy of the procedure. Placing the implants exactly where they were virtually planned provides a lot of clinical benefits [2,3]. Clinicians can avoid the anatomical structures which should not be invaded, i.e., the mandibular canal or the mental foramen, and it becomes possible to deliver the prefabricated prosthesis according to the virtually placed implant position immediately after the surgery.

Free hand implant placement, which has been widely performed for decades since dental implants were first introduced, has shown high survival rates; however, there are some disadvantages, such as higher dependence on the clinician’s experience and risk of a severe deviation of the actually placed implant position compared to the position planned prior to the surgery [4,5]. As digital dentistry based on computer-aided design/computer-aided manufacturing (CAD/CAM) has been proposed for the field of dental implantology, an enormous change has taken place. Impression taking with a rubber or silicone material and fabrication of the model with a dental stone have been replaced by an optically scanned image [6] which is superimposed with a computed tomography scan to virtually plan implant surgery and design a surgical template using computer software [7], and three-dimensional (3D) fabrication of a surgical template has made it possible to place implants as accurately as possible based on virtual planning by fully guided implant surgery [8]. It has eventually shortened the procedure time and relieved the patients’ discomfort and clinicians’ difficulty [9,10,11]. On the other hand, patients need multiple visits before the surgery to undergo computed tomography and optical impression by intraoral scanning, and it takes 1–2 days to design and fabricate the surgical template based on virtual implant planning.

Besides the most commonly used method mentioned above, a new type of fully guided implant surgery systems, VARO Guide^®^, has recently been proposed, which can be referred to as an “in-house model-free and zero-setup” surgical template. This system uses a unique intraoral device named “preguide” which resembles a single sextant-sized bite impression tray which contains light-cured composite resin. The plasticity of the pre-cured composite resin of the preguide enables clinicians to record the occlusion and take the impression of the edentulous sextant. The light-cured preguide is used for taking computed tomography scans. After loading radiographic images to planning computer software, the fully guided surgical guide is fabricated by milling of the post-cured preguide depending on the virtually planned implant position. Since this whole process, which takes less than an hour at chairside (in-house), does not involve either conventional or digital (model-free) model fabrication as well as laboratory work to fabricate the guide either manually of by 3D printing (zero-setup), it could be assumed that the present novel guide system would have a smaller chance of error and eventually show higher accuracy compared to the virtually designed and 3D-printed surgical template. There is, however, no research conducted to directly compare the two different types of surgical guide.

This in vitro study mainly aimed to compare VARO Guide^®^ to the surgical guide fabricated by CAD/CAM (NAVI Guide^®^) in terms of accuracy and efficacy of the implant surgery performed in the dentiform model. The null hypothesis of this experiment was that VARO Guide would show neither less positional deviation in implant placement nor shorter procedure time compared to NAVI Guide. The method proposed by a number of previous publications was used to evaluate and visualize how much discrepancy occurred between the virtually planned and actually placed positions of the implant by superimposing the STL file obtained at different timepoints [12,13].

## 2. Materials and Methods

### 2.1. Study Design and Group Allocation

The total of 20 dentists who worked at Yonsei University Dental Hospital participated in this experiment, and they were assigned to one of the following two groups:

- Expert group (*n* = 10): experience of implant surgery >100 cases
- Beginner group (*n* = 10): experience of implant surgery < 10cases

The demographic information of the experiment participants is summarized in Table 1.

### 2.2. Experimental Materials

#### 2.2.1. Surgical Guides

**Figure 1:**
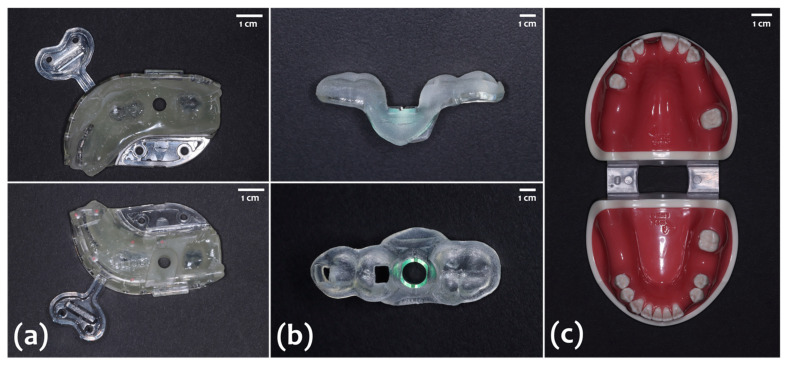
(**a**) VARO Guide^®^. (**b**) Navi Guide^®^. (**c**) The photograph of the dentiform model used in this experiment.

Two different types of surgical guide were prepared for the experiment:

- VARO Guide^®^ (Neobiotech, Seoul, Korea): a novel type of in-house, model-free and zero-setup surgical templates, which is fabricated by milling the light-cured preguide filled with composite resin composed of dimethacrylate and diurethane (Figure 1a)
- Navi Guide^®^ (Neobiotech): a polymethylmethacrylate (PMMA)-based surgical template with a metal sleeve, which is 3D-printed based on the virtual design (Figure 1b).

These two surgical guides were used for the following procedures:

- VG group: fully guided implant surgery using VARO Guide^®^ (VG);
- NG group: fully guided implant surgery using Navi Guide^®^ (NG).

For both surgical guide systems, 1-hour-long course of hands-on training with a dummy model was provided to all the research participants prior to the experiment.

#### 2.2.2. Dentiform Model and Implant Fixture

A total of 40 dentiform models of the lower jaw, of which the alveolar bone portion was made of resin and the gingiva portion was made of silicone, were used for the experiment (Figure 1c). The model had a single edentulous site in the left mandibular first molar area with adjacent teeth on mesial and distal surfaces. Twenty models were allocated to the VG and NG groups each, and an SLA-surfaced bone-level implant fixture (IS III, Neobiotech), 4.0 mm in diameter and 10 mm in length, was placed in the edentulous area by fully guided implant surgery.

### 2.3. Treatment Protocol

**Figure 2:**
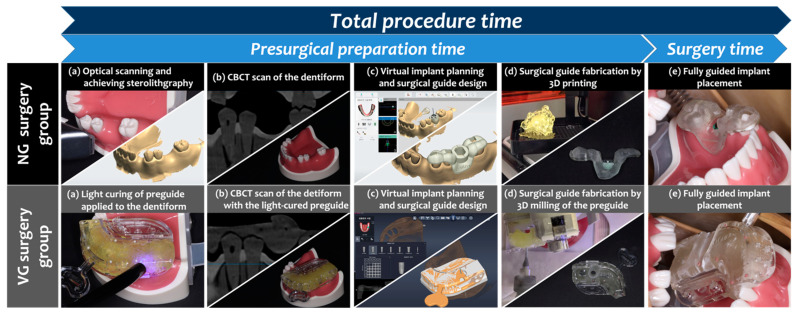
Complete workflows of the treatment procedures in the NG and VG groups.

To mimic the clinical situation as much as possible, all of the presurgical and surgical procedures (Figure 2) were conducted on a dental phantom manikin with the dentiform model fixed.

#### 2.3.1. Presurgical Virtual Implant Placement and Surgical Guide Fabrication for the VG Group

The preguide (PGM13, Neobiotech) containing a composite resin not yet polymerized was applied to the lower left posterior area, and the lower jaw was closed to take a centric occlusion bite impression. According to the manufacturer’s manual, the buccal and lingual sides of the preguide were light-cured intraorally for 15 s on each side at the wavelength of 470 nm and the power of 1000 mW/cm^2^ (DTE LUX-E Plus, MCDental, Blackpool, Lancashire, UK). Then, it was removed from the jaw, and the inner surface of the preguide was light-cured for 30 s. After repositioning the preguide to the dentiform model, cone beam computed tomography (CBCT; Q-FACE, HDX WILL, Seoul, Korea) was conducted (85 kV, 8 mA and the exposure time of 24 s) for both the upper and lower jaws.

The obtained CBCT images were transferred to computer software (VARO Plan, Neobiotech) for presurgical planning and surgical guide design, and the implant fixture was virtually placed in a prosthetically ideal position. Based on this position and the fixture size, the drilling hole was designed, and the design file was extracted from the software and transferred to a milling machine (VARO Mill, Neobiotech). As there were six radiopaque points surrounding the edge of the preguide, the milling machine recognized the location of the drilling hole, and the preguide loaded to the milling machine was converted to VG.

#### 2.3.2. Presurgical Virtual Implant Placement and Surgical Guide Fabrication for the NG Group

CBCT scans obtained by the same machine used for the VG surgery group and the optical impression taken by an intraoral scanner (Trios, 3Shape, Copenhagen, Denmark) were superimposed and then transferred to computer software (Implant Studio, 3Shape) for virtual implant placing in a prosthetically ideal position. The surgical guide was also designed and then sent to an outsourcing dental laboratory, and a total of 20 NG with a metal sleeve were 3D-printed (Objet30 Dental Prime, Stratasys, MN, USA) and delivered to the experiment participants after 6 h.

#### 2.3.3. Fully Guided Implant Surgery

Both VG and NG surgeries were conducted flapless, with the surgical kits oriented for VG and NG, respectively. After applying the soft tissue punch to remove the mucosa, sequential drilling as well as countersinking of the cortical bone was driven, and the implant fixture was installed depending on the surgical guide. Following the implant surgery, a scan body, a resin-based abutment-looking device, was applied over the implant fixture, and the optical impression of the left lower posterior area was taken with an intraoral scanner to generate postsurgical stereolithography (STL).

It was randomly determined which of the research participants would perform one of the two surgeries first according to the random sequence generated with a four-block size using an online statistical computing program (http://www.sealedenvelope.com, accessed on 16 December 2019). To minimize the influence of the previous surgery experience on the next surgery, the interval between the VG and NG surgeries was set to two weeks for every participant.

### 2.4. Outcome Variables and Measurements

#### 2.4.1. Accuracy of Implant Placement

To compare the virtually planned position to the actually placed position, presurgical planning data were first superimposed to the postsurgical STL files. Since two different computer applications were used for the VG and NG groups, superimposition and analysis were carried out differently as well.

For the VG group, CAM mesh data of the guide design collected from the VG planning software (VARO Plan, Neobiotech) and presurgical CBCT scans were prepared and superimposed using dental CAD software (exoCAD, exocad GmbH, Darmstadt, Germany). The postsurgical STL file obtained in the VG surgery group was also uploaded to the same software, and the scan body was converted to a virtual abutment. For the NG group, the project file was extracted from the planning software (Implant Studio, 3Shape). Then, it was uploaded to CAD software (Dental System, 3Shape) from the same company that developed the planning software used for the NG surgery, and the postsurgical STL file was uploaded to the same CAD software as well. An abutment, the same as the one virtually placed in the VG group, was designed over the presurgically planned implant and the scan body of the postsurgical STL file.

These presurgical and postsurgical datasets of the VG and NG groups were loaded in 3D inspection computer software (Geomagic Verify, SculptCAD, Dallas, TX, USA). The preoperative and postoperative datasets with a merged layer of the abutment design were superimposed to each other using adjacent teeth as references. In the VG group, the angle and the centers of the platform and the apex of the virtually planned implant were determined based on the depth and axis of the drilling hole which had been formed in the VG, while those of the actually placed implant were deduced using the reverse engineering technique as explained in the previous study [14], visualizing inversely from the design of the abutment. In the NG group, both presurgical and postsurgical locations of the angle and the centers of the platform and the apex of the fixture were determined using the reverse engineering technique.

Following parameters were measured for the assessment of the implant placement accuracy:

- Vertical deviation (mm): the linear deviation measured in the aspect of vertical height of the implant fixture platform.
- Angular deviation (°): the angle measured between the virtually planned axis and the actually placed axis of the implant.
- Platform deviation (mm): the linear deviation measured in the horizontal aspect at the implant fixture platform level.
- Apex deviation (mm): the linear deviation measured in the horizontal aspect at the implant fixture apex level.

**Figure 3:**
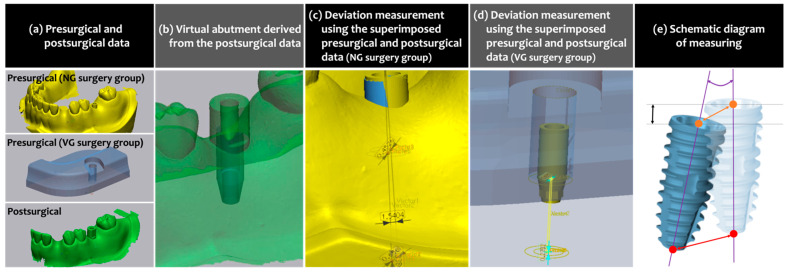
Accuracy of implant position measurements. (**a**) Presurgical and postsurgical data. The presurgical data of the NG and VG groups were extracted from the respective computer software, and the postsurgical data were obtained by optical impression taking following the scan body application in both groups. (**b**) The abutments virtually designed over the actually placed implant. (**c**,**d**) Measuring the deviation between the virtually planned and actually placed implants after superimposing the presurgical and postsurgical data. (**e**) Schematic diagram showing the method of measuring the deviation between the virtually planned (transparent fixture) and actually placed (vivid fixture) implants: vertical deviation (mm; black double-headed arrow) measured as the distance between two black parallel lines passing the center of the fixture platform; angular deviation (°; purple curve) between the purple lines representing the long axis of the implants; platform deviation (mm; orange double-headed arrow) between the orange dots showing the centerpoint of the fixture platform; apex deviation (mm: red double-headed arrow) between the red dots showing the centerpoint of the fixture apex.

The whole measuring process mentioned above is summarized in Figure 3.

#### 2.4.2. Procedure Time

The time spent on the VG and NG surgeries was measured by a single investigator (J.K.) with a stopwatch and estimated as follows:

- Total procedure time: the time spent from the beginning of the presurgical preparation to the end of the implant surgery.
- Presurgical preparation time: the time spent on the virtual implant planning and surgical guide fabrication estimated in detail as follows:
  - Curing/scanning time: time spent on preguide curing (VG group only) or optical scanning (NG group only);
  - Virtual planning time: time spent on virtual planning with the corresponding computer software of each surgery group;
  - Guide fabrication and delivery time: time spent from the start of milling (VG group only) or 3D printing (NG group only) to the delivery of the surgical guide to the surgeon.
- Surgery time: the time spent on the fully guided implant placement only (from the start of the surgical guide sitting onto the dentiform model to the final installation of the implant fixture).

### 2.5. Statistical Analysis

All the data were statistically analyzed using computer software (SPSS, version 23, IBM, Armonk, NY, USA). Due to the small sample size, nonparametric comparison was conducted, and the Mann–Whitney *U* test was performed to compare the outcomes as follows:

- VG group versus NG group, in terms of implant placement accuracy and procedure time.
- VG group versus NG group within the expert group, in terms of implant placement accuracy and procedure time.
- VG group versus NG group within the beginner group, in terms of implant placement accuracy and procedure time.
- Expert group versus beginner group within the VG group, in terms of implant placement accuracy and procedure time.
- Expert group versus beginner group within the NG group, in terms of implant placement accuracy and procedure time.

All the data were represented by the mean, the median and the 95% confidence intervals, and statistical significance was set as *p* < 0.05.

## 3. Results

### 3.1. Accuracy of Implant Placement

**Figure 4:**
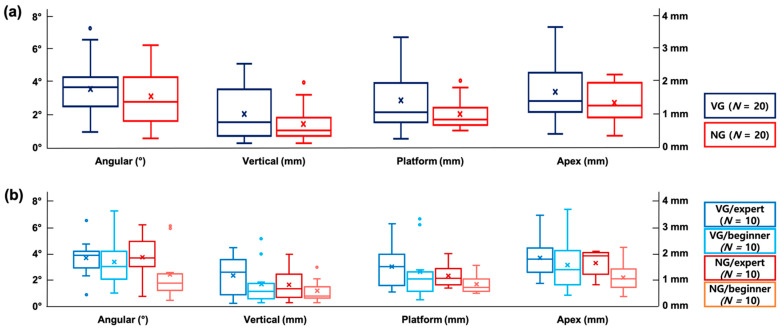
Box-and-whisker plots showing positional accuracy of the implant fixtures. (**a**) VG surgery group versus NG surgery group. (**b**) Expert group versus beginner group within VG surgery and NG surgery each; VG surgery group versus NG surgery group within expert group and beginner group each. The whiskers above and below the box show the minimum and the maximum, respectively, and the upper and lower bars of each box represent the first and third quartiles, respectively. The horizontal line within each bar means the median value, while the “x” mark denotes the mean value. The circular points placed above and below the whiskers depict the outliers if applicable.

The data on implant placement accuracy are summarized in Figure 4 and Table 2.

#### 3.1.1. VG versus NG Surgery

The vertical, angular, platform and apex deviations (mean (median)) in the VG surgery (0.95 (0.71) mm, 3.49° (3.62°), 1.37 (1.01) mm and 1.68 (1.41) mm, respectively) were larger than those in the NG surgery (0.64 (0.44) mm, 3.05° (2.69°), 0.95 (0.78) mm and 1.34 (1.25) mm, respectively); however, there was no statistical significance (*p* > 0.05).

#### 3.1.2. VG versus NG Surgery within the Expert and Beginner Groups

Within the expert group, the vertical, platform and apex deviations in the VG surgery (1.12 (1.24) mm, 1.48 (1.47) mm and 1.81 (1.77) mm, respectively) were slightly larger than those in the NG surgery (0.75 (0.62) mm, 1.12 (1.01) mm and 1.62 (1.92) mm, respectively), whereas the angular deviation was slightly lower in the VG surgery (3.64° (3.86°)) than in the NG surgery (3.72° (3.64°)). In the beginner group, the VG surgery showed larger deviations in the vertical, angular, platform and apex deviations (0.78 (0.51) mm, 3.34° (2.97°), 1.25 (0.98) mm and 1.54 (1.36) mm, respectively) compared to the NG surgery (0.52 (0.33) mm, 2.37° (1.71°), 0.78 (0.66) mm and 1.07 (1.02) mm, respectively). None of the differences, however, demonstrated statistical significance (*p* > 0.05).

#### 3.1.3. Expert Group versus Beginner Group within the VG and NG Surgery

In the case of the VG surgery, the expert group featured larger vertical, angular, platform and apex deviations (1.12 (1.24) mm, 3.64° (3.86°), 1.48 (1.47) mm and 1.81 (1.77) mm, respectively) than the beginner group (0.78 (0.51) mm, 3.34° (2.97°), 1.25 (0.98) mm and 1.54 (1.36) mm, respectively). The NG surgery also showed larger deviations in all the aspects (vertical, angular, platform and apex) in the expert group (0.75 (0.62) mm, 3.72° (3.64°), 1.12 (1.01) mm and 1.62 (1.92) mm, respectively) compared to those in the beginner group (0.52 (0.33) mm, 2.37° (1.71°), 0.78 (0.66) mm and 1.07 (1.02) mm, respectively). All of the differences, however, were not statistically significant (*p* > 0.05).

### 3.2. Procedure Time

The procedure time measured is summarized in Table 3 and Table 4.

#### 3.2.1. VG versus NG Surgery

The total time spent on the VG surgery (26.33 (28.58) min) was significantly shorter than that spent on the NG surgery (378.83 (379.35) min; *p* < 0.05). The presurgical preparation time was significantly shorter in the VG surgery (19.63 (20.93) min) compared to the NG surgery (372.93 (372.95) min; *p* < 0.05), while the time needed for implant placement was not significantly different between the VG and NG surgery (6.70 (7.07) min and 5.90 (5.88) min, respectively; *p* > 0.05).

When the presurgical preparation time was compared in more detail, the time spent on preguide curing (VG group) or optical scanning (NG group) was similar to each other; however, it took significantly longer in the NG group to virtually plan with the respective computer software, fabricate and deliver the guide to the surgeons (9.52 (8.30) min and 360.00 (360.00) min, respectively) compared to the VG group (5.68 (5.73) min and 10.75 (12.07) min, respectively; *p* < 0.05).

#### 3.2.2. VG versus NG Surgery within the Expert and Beginner Groups

Both the expert group and beginner group participants spent significantly less total procedure time on the VG surgery (28.30 (29.37) min and 24.37 (25.55) min, respectively) compared to the NG surgery (376.68 (377.00) min and 380.98 (380.93) min, respectively; *p* < 0.05). Similarly, the time spent on the preparation of the VG surgery was significantly shorter in both groups (21.68 (22.80) min in the expert group and 17.06 (16.75) min in the beginner group) than that spent on the NG surgery (371.88 (371.72) min in the expert group and 373.97 (374.92) min in the beginner group; *p* < 0.05). The surgical time, however, was comparable between the VG and NG surgery in both groups (*p* > 0.05).

Regarding the presurgical preparation time, the time spent on curing the VG preguide and for optical scanning to design NG was comparable in both the expert and beginner groups (*p* > 0.05). It took a similar time for the experts to virtually plan VG and NG surgeries using the respective computer software (*p* > 0.05), whereas the beginners needed significantly less time for planning the VG surgery (3.78 (3.28) min) compared to the NG surgery (10.90 (11.73) min; *p* < 0.05). Regardless of the participants’ surgical experience, significantly less time was needed for fabricating and delivering VG (11.13 (12.10) min in the expert group; 10.37 (10.87) min in the beginner group) when compared to the times needed for NG (360.00 (360.00) min for both the experts and beginners; *p* < 0.05).

#### 3.2.3. Expert Group versus Beginner Group within the VG and NG Surgery

The total time spent in the VG and NG surgery each was comparable between the expert and beginner groups (*p* > 0.05). The expert group needed a significantly longer time for preparing for the VG surgery than the beginner group did (21.68 (22.80) min vs. 17.06 (16.75) min, respectively; *p* < 0.05), but for preparing for the NG surgery, the time spent by the expert and beginner groups was similar (*p* > 0.05). Regarding the surgery time, the expert group completed implant placement in the NG surgery group significantly faster than the beginner group (4.80 (4.92) min vs. 7.02 (6.57) min; *p* < 0.05), whereas the time needed for implant placement in the VG surgery group was comparable between the expert and beginner groups.

When the preparation time was analyzed in more detail, the experts spent significantly longer time in virtual planning of the VG surgery using computer software compared to the beginners (7.58 (7.88) min vs. 3.78 (3.28) mm; *p* < 0.05) while neither the curing/scanning time nor guide fabrication and delivery time were not significantly different (*p* > 0.05). In the case of the NG surgery, both the experts and the beginners spent a similar time (*p* > 0.05).

## 4. Discussion

This experiment investigated positional accuracy of an implant placed in a plastic dentiform model using two different surgical guide systems, model-free and zero-setup VG and 3D-printed and metal-sleeved NG. The mean angular and linear (vertical, platform and apex) deviations were comparable between the two guide systems, which were less than 3.5° and 2 mm, respectively. The total time spent on the VG surgery was approximately 6 h shorter than that spent on the NG surgery, which was mainly due to the significant time difference in presurgical preparation, but the implant placement time per se did not differ between the VG and NG surgery. The proficiency and experience in implant surgery did not seem to affect implant placement accuracy in both the VG and NG surgery. The total procedure time was also similar between the expert or beginner groups; however, the presurgical preparation time for the VG surgery and the surgical time for the NG surgery were significantly shorter in the beginner group and the expert group, respectively.

The accuracy of both VG and NG in the present study seemed reliable when compared to the outcomes from the previous experiments [13,14,15,16] which reported that stereolithographic surgical guides caused angular and linear deviations ranging between 3° and 4° and 1 mm and 2 mm on average, respectively. Furthermore, a recent systematic review and a meta-analysis of 20 randomized clinical trials revealed that the total mean linear deviation of 1.2 mm (platform level) and 1.4 mm (apex level) and the total mean angular deviation of 3.5° [17] were similar to the ones observed in this study. This amount of error is considered appropriate to avoid the invasion of critical anatomical structures (i.e., mandibular canal, mental foramen or maxillary sinus floor) since the safety margin for anatomic consideration was reported as an error of less than 2 mm [17]. Meanwhile, it is insufficient in terms of passively delivering the prefabricated prosthesis for immediate provisionalization or loading as the clinically acceptable range of error for prosthetic fitting is known to be from 0.05 mm to 0.15 mm [18,19,20]. This means that both VG and NG may provide a benefit to clinicians to conduct a safe surgery, but they need more improvement to let them deliver the prosthesis designed and fabricated at the presurgical planning stage.

The use of stereolithographic surgical templates for fully guided implant placement has been providing advantages to both clinicians and patients in the aspect of convenience and accuracy; however, still, there has been a concern in having a possibility of error during the registration of the scan data obtained by either intraoral scanning or cast model scanning with CBCT images [21,22]. Furthermore, it has been reported that though both the milling and 3D printing methods showed reliable accuracy within the safety margin, the milling procedure caused less deviation in fabricating the guide when compared to 3D printing [23].

In this vein, VG seems to have a benefit over 3D-printed templates such as NG. The preguide with indentations, which serves as a bite impression, is applied intraorally for conducting CBCT without intraoral scanning or making a cast model, and it is directly converted to the final surgical guide by drilling a hole with a milling machine. It eventually decreases the chance of error possibly caused by superimposition of the data and 3D printing [24]. In addition, there is no need for an additional appointment for the patient to check whether the fabricated template fits well or not as the bite indentations of the adjacent teeth contribute to passive sitting. Despite these potential benefits of VG, superiority in terms of positional accuracy over NG was not found. This might have been due to the fact that VG were unfamiliar to the experiment participants, whereas NG had similar features to the other various guide systems and were more familiar. In this experiment, all of the participants conducted the VG surgery in the dentiform model for the first time following 1-hour-long hands-on training. It could be considered interesting that the VG surgery showed an acceptable extent of accuracy in spite of the limited time for getting used to the guide system; however, given that there have been studies reporting the presence of the learning curve [25,26], it could be assumed that positional accuracy of the implants placed using VG might be improved after repeated surgeries.

Even though the accuracy of the beginner group was slightly higher than that of the expert group in both VG and NG surgery, the difference was not statistically significant. This implies that VG as well as NG can be properly used by any clinicians after appropriate training. Meanwhile, more outlier data were found in the beginner group (angular, vertical and platform deviations) regardless of the guide system. On the other hand, the expert group featured outlier data in the angular deviation after the VG surgery only, which means the expert group showed more consistency compared to the beginner group. This could be explained by the difference in clinical experience between the two participant groups. There were a few beginners who caused larger deviation than the others who showed better performance. Though there were outliers in terms of angular deviation in the expert group as well, it seems that the experts got used to the novel and unfamiliar guide system faster than the beginners owing to their experience in the typical type of guide systems, which resulted in more consistent outcomes.

In terms of the total procedure time, the VG surgery required significantly shorter time than the NG surgery did. This was obviously due to the difference in the time spent on presurgical preparation, and more specific, significant differences were found in the time needed for software-aided virtual planning and surgical guide delivery after milling/3D printing. On the contrary, the time spent solely on implant placement was similar in both the VG and NG groups. Considering that the computer software for the VG surgery planning was novel to all the participants and therefore less familiar to the experiment participants when compared to the one used for the NG surgery, it could be considered that the VG surgery planning software is not difficult to learn and use even after a short period of training. A huge difference in the time needed for guide fabrication and delivery was caused by the fact that VG milling took place at chairside while NG were 3D-printed at an outsourcing dental laboratory and took 6 h to be delivered to the clinicians. These outcomes imply that the benefit of VG in terms of time effectiveness mostly lies in the convenience of using planning software and the chairside milling process. If a 3D printer is available at the clinic, NG could also be immediately delivered to the clinician; however, given that 3D printers strictly require air conditioning and ventilation equipment, it is not easy to 3D-print the surgical guide at chairside at present.

Even though the total time that the experts and the beginners spent on the VG and NG surgery was comparable in both instances, interestingly, there were significant differences between the expert and beginner groups in the time needed to prepare for the VG surgery and to place the implant in the NG surgery. These findings are considered to be owing to the difference in the degree of familiarity with working with planning software and guide surgery kits. Given that the mean age of the beginner group participants was younger than that of the expert group ones, they seemed to be prone to learn how to use new VG surgery CAD/CAM software easier and faster than the experts. Moreover, the be-ginners spent significantly less time when they used the VG surgery software than when they used the NG surgery software. In the similar vein, the reason why the experts spent less implant placing time in the NG surgery seemed to lie in the fact that the experts had a lot more experience in regular fully guided implant surgery compared to the beginners.

In this experiment, only tooth-supported single edentulous cases were chosen to solely compare the two different guide systems without the interference of any other factors; however, it could be considered as one of the limitations, as well the nature of an in vitro study using a plastic dentiform model. Since there is a lot of factors affecting the accuracy of implant placement, such as the type of jaw or the way of guide supporting [14] as well as the learning curve [25,26], the findings of this study need to be further verified in the preclinical and clinical stages in various situations.

## 5. Conclusions

Regardless of the surgeons’ proficiency, both the VG and NG systems demonstrated reliable performance in implant placement accuracy to a similar extent to each other. The total time spent on the VG surgery was, however, significantly shorter than that spent on the NG surgery, which resulted from the significant difference between the VG and NG surgery in terms of the presurgical preparation time. Despite the implant placement accuracy of VG was not superior to that of NG, it could be implied that VG have a potential to provide a clinical benefit to both the surgeons and patients by shortening the time from the treatment planning to the completion of the implant surgery.

## Figures and Tables

**Table 1:** Demographic information of the experiment participants.

	Overall (Experts + Beginners)	Expert Group	Beginner Group
Number of participants	20	10	10
Age (mean ± standard deviation, years)	29.55 ± 3.44	31.2 ± 3.29	27.9 ± 3.07
Period of dental implant surgeon career (mean ± standard deviation, years)	3.10 ± 2.29	4.80 ± 2.10	1.40 ± 0.52

**Table 2:** Accuracy analysis of the implant position (mean, median (95% confidence interval)).

Groups		Vertical Deviation (mm)	Angular Deviation (°)	Platform Deviation (mm)	Apex Deviation (mm)
VG surgery group	Overall (experts + beginners)	0.95, 0.71 (−0.60, 1.30)	3.49, 3.62 (2.73, 4.25)	1.37, 1.01 (0.95, 1.79)	1.68, 1.41 (1.28, 2.08)
	Expert group (*N* = 10)	1.12, 1.24 (0.64, 1.60)	3.64, 3.86 (2.70, 4.58)	1.48, 1.47 (0.96, 2.01)	1.81, 1.77 (1.34, 2.29)
	Beginner group (*N* = 10)	0.78, 0.51 (0.27, 1.29)	3.34, 2.97 (2.11, 4.58)	1.25, 0.98 (0.58, 1.93)	1.54, 1.36 (0.89, 2.20)
NG surgery group	Overall (experts + beginners)	0.64, 0.44 (0.40, 0.88)	3.04, 2.69 (2.19, 3.90)	0.95, 0.78 (0.75, 1.14)	1.34, 1.25 (1.08, 1.60)
	Expert group (*N* = 10)	0.75, 0.62 (0.36, 1.15)	3.72, 3.64 (2.66, 4.78)	1.12, 1.01 (0.83, 1.42)	1.62, 1.92 (1.30, 1.93)
	Beginner group (*N* = 10)	0.52, 0.33 (0.26, 0.79)	2.37, 1.71 (1.12, 3.62)	0.78, 0.66 (0.55, 1.00)	1.07, 1.02 (0.72, 1.42)

**Table 3:** Procedure time (mean, median (95% confidence interval)).

Groups		Total Time (min)	Presurgical Preparation Time (min)	Surgery Time (min)
VG surgery group	Overall (experts + beginners)	26.33, 28.58 (23.57, 29.10)	19.63, 20.93 (17.52, 21.75)	6.70, 7.07 (5.70, 7.68)
	Expert group (*N* = 10)	28.30, 29.37 (24.27, 32.33)	21.68, 22.80 (19.00, 24.35)	6.62, 7.40 (5.10, 8.15)
	Beginner group (*N* = 10)	24.37, 25.55 (20.82, 27.92)	17.06, 16.75 (14.72, 20.48) +	6.77, 6.13 (5.42, 8.12)
NG surgery group	Overall (experts + beginners)	378.83, 379.35 (376.57, 381.10) *	372.93, 372.95 (371.07, 374.78) *	5.90, 5.88 (4.97, 6.83)
	Expert group (*N* = 10)	376.68, 377.00 (373.85, 379.52) *	371.88, 371.72 (369.40, 374.37) *	4.80, 4.92 (3.68, 5.92)
	Beginner group (*N* = 10)	380.98, 380.93 (377.88, 384.08) *	373.97, 374.92 (371.25, 376.70) *	7.02, 6.57 (5.83, 8.18) +

Note: * significantly different from the VG surgery group when compared overall, within the expert group and the beginner group (*p* < 0.05); + significantly different from the expert group when compared within the VG surgery group and the NG surgery group (*p* < 0.05).

**Table 4:** Presurgical preparation time in detail (mean, median (95% confidence interval)).

Groups		Curing/Scanning Time (min)	Virtual Planning Time (min)	Guide Fabrication and Delivery Time (min)
VG surgery group	Overall (experts + beginners)	3.22, 3.40 (2.83, 3.58)	5.68, 5.73 (4.53, 6.82)	10.75, 12.07 (9.63, 11.87)
	Expert group (*N* = 10)	2.97, 3.30 (2.47, 3.45)	7.58, 7.88 (6.33, 8.83)	11.13, 12.10 (9.66, 12.60)
	Beginner group (*N* = 10)	3.47, 3.40 (2.93, 4.00)	3.78, 3.28 (2.85, 4.72) +	10.37, 10.87 (8.63, 12.08)
NG surgery group	Overall (experts + beginners)	3.42, 3.10 (2.97, 3.87)	9.52, 8.30 (7.68, 11.35) *	360.00, 360.00 (360.00, 360.00) ^a,^*
	Expert group (*N* = 10)	3.75, 3.68 (2.95, 4.55)	8.13, 7.66 (5.75, 10.52)	
	Beginner group (*N* = 10)	3.08, 3.10 (2.73, 3.42)	10.90, 11.73 (8.27, 13.53) *	

Note: ^a^ all NG required the same amount of time (6 h) to be fabricated and delivered; * significantly different from the VG surgery group when compared overall, within the expert group and the beginner group (*p* < 0.05); + significantly different from the expert group when compared within the VG surgery group and the NG surgery group (*p* < 0.05).

## Data Availability

All the data are contained within the article.
